# Ecological implications of single and mixed nitrogen nutrition in *Arabidopsis thaliana*

**DOI:** 10.1186/1472-6785-13-28

**Published:** 2013-07-23

**Authors:** Gordon G McNickle, Michael K Deyholos, James F Cahill Jr

**Affiliations:** 1 Department of Biological Sciences, University of Alberta, CW405, Edmonton, AB, T6G 2E9, Canada; 2 Current address: Department of Biology, Wilfrid Laurier University, 75 University Avenue West, Waterloo, ON N2L 3C5, Canada

**Keywords:** Amino acid uptake, *Arabidopsis thaliana*, Reproductive output, Nitrogen partitioning, Nitrogen preferences, Plant foraging

## Abstract

**Background:**

Ecologists recognize that plants capture nitrogen in many chemical forms that include amino acids. Access to multiple nitrogen types in plant communities has been argued to enhance plant performance, access to nitrogen and alter ecological interactions in ways that may promote species coexistence. However, data supporting these arguments have been limited. While it is known that plants uptake amino acids from soil, long term studies that link amino acid uptake to measures of plant performance and potential reproductive effort are not typically performed. Here, a series of experiments that link uptake of nitrate, glutamine or asparagine with lifetime reproductive effort in *Arabidopsis thaliana* are reported. Nitrogen was offered either singly or in mixture and at a variety of combinations. Traits related to reproductive output were measured, as was the preference for each type of nitrogen.

**Results:**

When plants were supplied with a single nitrogen type at concentrations from 0.1-0.9 mM, the ranking of nitrogen types was nitrate > glutamine > asparagine in terms of the relative performance of plants. When plants were supplied with two types of nitrogen in mixture at ratios between 0.1:0.9-0.9:0.1 mM, again plants performed best when nitrate was present, and poorly when amino acids were mixed. Additionally, stable isotopes revealed that plants preferentially captured nitrogen types matching the hierarchy of nitrate > glutamine > asparagine. Comparing between the two experiments revealed that mixed nitrogen nutrition was a net cost to the plants.

**Conclusions:**

Plant performance on mixed nitrogen was less than half the performance on equal amounts of any single nitrogen type. We asked: why did *A. thaliana* capture amino acids when doing so resulted in a net cost? We argue that available data cannot yet answer this question, but hypothesize that access to lower quality forms of nitrogen may become important when plants compete.

## Background

Plant ecologists increasingly recognize that plants can capture nitrogen in a variety of different chemical forms, ranging from inorganic forms such as nitrate and ammonium to as many as 20 different amino acids [1-6]. This access to organic pools of nitrogen has been argued to increase plant access to nitrogen, especially in nitrogen limited habitats E.g. [1,2,6-8]. Additionally, ecologists have argued that plant access to a variety of different forms of nitrogen may also influence population and community processes by allowing increased dimensions of niche partitioning mediated through differences in nitrogen preference [7,9-13]. However, while ecologists have obtained large amounts of data investigating whether plants can or cannot capture specific amino acids, there is surprisingly little data that links this uptake to plant growth and performance. We argue that it is not enough to simply show that plants will use certain forms of nitrogen, but for ideas surrounding potential ecological dynamics we need data that show these different types of nitrogen are actually of value to plants.

Generally, uptake rates for amino acids are considerably lower compared to inorganic forms of nitrogen [1,3,4,14], though they may be higher [6,15]. Data suggests that, when grown with amino acids as the sole nitrogen source, the model plant *Arabidopsis thaliana* ((L.) Heynh, var. Columbia) was only capable of achieving between 1% and 50% of its potential vegetative growth on an identical concentration of nitrate [4]. In general it appears that most of the amino acids that plants can use as a source of nitrogen depress plant growth relative to mineral sources of nitrogen [1,16] while only a minority of amino acids can occasionally increase plant growth [6,16] or at least match plant growth on mineral forms of nitrogen [17]. However, almost all the available data come from single nitrogen nutrition studies, and multiple nitrogen types in mixture are rarely offered to plants. Yet, plants in ecological communities will experience soil with multiple nitrogen types available simultaneously, and it is important to understand the nature of any interactions among nitrogen types. This means that plants may have to select among nitrogen types, and that there may also be positive or negative interactions among nitrogen types in their contribution to plant performance.

In this paper, three experiments are presented which were designed to explore the consequences of single and mixed nitrogen nutrition for the growth and potential reproductive output of the model plant *Arabidopsis thaliana* ((L.) Heynh, var. columbia). The goal was to explore links between mixed nitrogen nutrition and plant growth and reproductive output. In experiment 1, correlations between traits measured during the vegetative growth phase and lifetime reproductive effort at senescence were examined. Here, the goal was to be able to estimate potential lifetime performance of individuals harvested prior to senescence. In experiment 2, plants were offered one of three types of nitrogen at nine concentrations and plant traits were measured to understand how single nitrogen nutrition influenced plant growth and reproductive output. This was done as a control, to examine plant performance on single nitrogen nutrition. In experiment 3, mixed nitrogen nutrition was examined by growing plants with two types of nitrogen simultaneously at 9 different ratios for all pairwise combinations of nitrate, glutamine and asparagine. The relative amounts of each form of nitrogen captured, and plant traits were each measured to determine how plant growth and reproductive effort varied on mixed nitrogen nutrition. It was hypothesized that plants would be capable of growing and reproducing when fed amino acids, but those amino acids would contribute relatively little to plant growth and reproduction compared to mineral sources of nitrate. It was also hypothesized that given two types of nitrogen; plants would be unlikely to preferentially capture amino acids. At the end of the manuscript, we return to ideas about ecology and evolution and suggest a closer link between questions asked by plant ecologists working at all levels of organization would greatly advance the general understanding of amino acid nutrition in plant systems.

## Results

### Experiment 1: traits and reproductive effort

**Figure 1:**
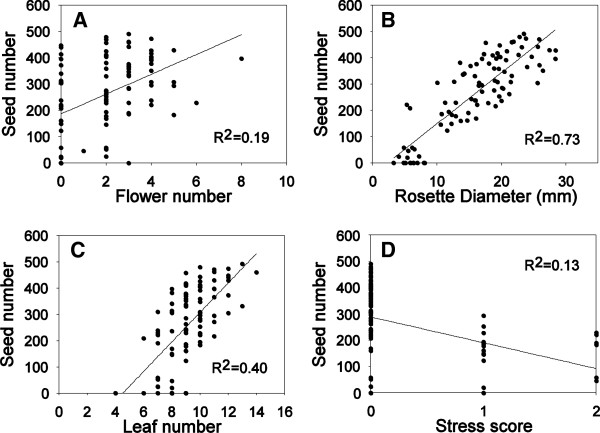
**Relationship between traits measured at 4 weeks of age and seed production at senescence (10 weeks) in experiment 1. (A)** Flower number, **(B)** rosette diameter, **(C)** leaf number and **(D)** stress score.

In experiment 1, plants were grown at a range of different nutrient availabilities to generate variability in size, and values of plant traits in the middle of their growth cycle (4 weeks of age) were linked to lifetime seed production at senescence (10 weeks of age) through regression. We measured leaf number, rosette diameter, flower number and scored plant stress based on leaf colour. Leaf number (F_1,91_ = 61.9, p < 0.0001 , R^2^ = 0.40), rosette diameter (F_1,91_ = 254.9, p < 0.0001 , R^2^ = 0.73) and flower number (F_1,91_ = 22.1, p < 0.0001 , R^2^ = 0.19) at 4 weeks of age were all positively correlated with lifetime seed production (Figure 1A-C). Physiological stress (F_1,91_ = 15.2, p = 0.0002, R^2^ = 0.13) was negatively correlated with lifetime seed production indicating that high nutrient stress was generally related to low reproductive output (Figure 1D). Though all traits were significantly correlated with lifetime seed production, rosette diameter at 4 weeks of age was the most efficient predictor of lifetime seed production at senescence (Figure 1B). This analysis allows us to link vegetative growth traits to potential reproductive output in plants.

### Experiment 2: Single nitrogen nutrition

**Figure 2:**
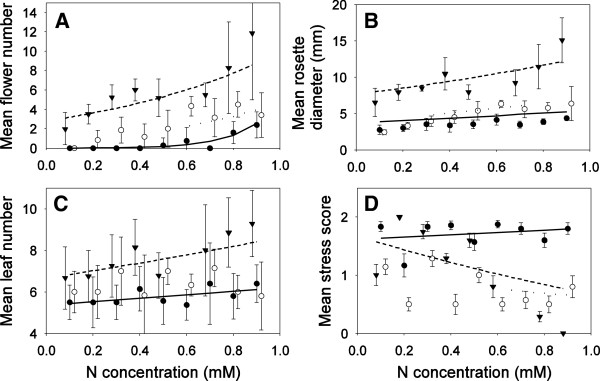
**Relationship between nitrogen availability (mM) and mean plant traits at 4 weeks of age from experiment 2 (1 standard deviation is shown).** The lines are fitted lines from the GLMs (Table 2). In all panels, nitrate (Filled triangles, dashed line), glutamine (open circles, solid line) and asparagine (filled circles, dotted line). **(A)** Flower number, **(B)** rosette diameter, **(C)** number of leaves, and **(D)** stress index. Note that count data was fit with a Poisson error distribution and a log link function, which can produce the appearance of non-linear fits in the untransformed scale.

In this experiment plants were grown with only one type of nitrogen (nitrate, glutamine or asparagine) at nine different concentrations (0.1-0.9 mM in 0.1 mM increments) to investigate the potential for each nitrogen type alone to contribute to plant growth and reproductive output. Data indicate that plants were able to grow and produce flowers on all nitrogen types confirming that nitrate, glutamine and asparagine were each viable sole sources of nitrogen for *A. thaliana* (Figure 2). However, the three types of nitrogen had significantly different effects on plant performance (Table 1); in general, plants performed best by any metric on nitrate, intermediate on glutamine and poorest on asparagine (Figure 2). Although plants could grow and reproduce with asparagine as the sole source of nitrogen (Figure 2A), the plants were often small (rosettes < 5 mm in diameter, Figure 2B), had few flowers (median = 0, max = 3, Figure 2A) and were obviously nutrient stressed (Figure 2D). For any of the three nitrogen types, the concentration of nitrogen was positively correlated with flower number and rosette diameter (Figure 2, Table 1). Within the narrow range of nitrogen concentrations used (0.1-0.9 mM), nutrient stress and leaf number were relatively invariant regardless of nitrogen concentration (Table 1). However, there was a marginally significant trend towards decreasing stress with increasing nitrate concentration compared to relatively constant stress for plants supplied either form of amino acid (Figure 2C, Table 1). The significant interaction between nitrogen type and concentration for flower number can be explained because glutamine and asparagine produced similarly low numbers of flowers at low concentrations causing the regression lines to cross at low concentrations (Figure 1A).

**Table 1:** Results of GLMs on plant traits by nitrogen type (nitrate, glutamine or asparagine), and nitrogen abundance in experiment 2

**Trait**	**Factor**	**Df**	**F**	**p**
Flower number	Nitrogen	2	31.9	<0.001
	Concentration	1	29.3	<0.001
	Nitrogen × Concentration	2	8.6	<0.001
	Residuals	166		
Rosette diameter	Nitrogen	2	38.5	<0.001
	Concentration	1	10.6	0.001
	Nitrogen × Concentration	2	1.5	0.211
	Residuals	166		
Leaf number	Nitrogen	2	3.4	0.037
	Concentration	1	1.6	0.214
	Nitrogen × Concentration	2	1.9	0.158
	Residuals	166		
Stress index	Nitrogen	2	3.4	0.037
	Concentration	1	0.2	0.65
	Nitrogen × Concentration	2	1.3	0.062
	Residuals	166		

### Experiment 3: mixed nitrogen nutrition

In this experiment plants were supplied with two types of nitrogen simultaneously, at nine different ratios (1:9, 2:8, 3:7, 4:6, 5:5, 6:4, 7:3, 8:2, 9:1) such that the total amount of nitrogen was always 1 mM. The goal was to investigate whether plants had any preferential uptake when offered two types of nitrogen at the same time, and to examine how mixed nitrogen nutrition influenced plant traits and reproductive effort relative to single nitrogen nutrition. We asked three questions: (i) did the plants capture amino acids intact? (ii) did the plants show any preferential uptake when offered two nitrogen types simultaneously? (iii) how did plant traits vary based on the ratios of nitrogen available?

**Figure 3:**
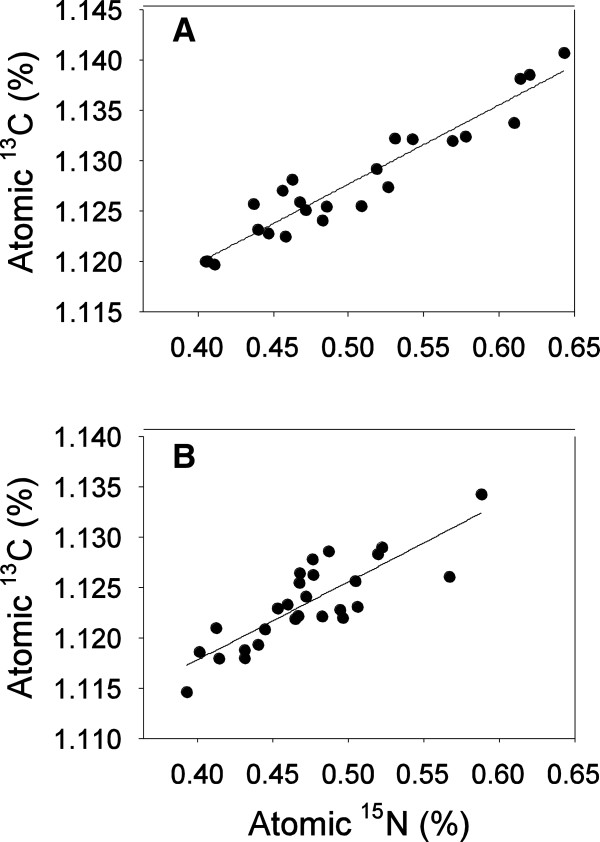
**Relationship between %15N and %13C captured by the plants for (A) glutamine uptake (from nitrate & glutamine treatment) and (B) asparagine uptake (from the nitrate & asparagine treatment).** Plants from the glutamine & asparagine treatment were too small to analyze for both %15N and %13C.

First, the data from dual labeled amino acids suggest amino acids were captured intact. The %^15^ N and %^13^C enrichment of each plant were compared and indeed, %^15^ N and %^13^C enrichment were strongly positively correlated for both glutamine uptake (F_1,22_ = 208.3, p < 0.0001 , R^2^ =0.90) and asparagine uptake (F_1,25_ = 57.62, p = 0.0024 , R^2^ =0.69) (Figure 3).

**Figure 4:**
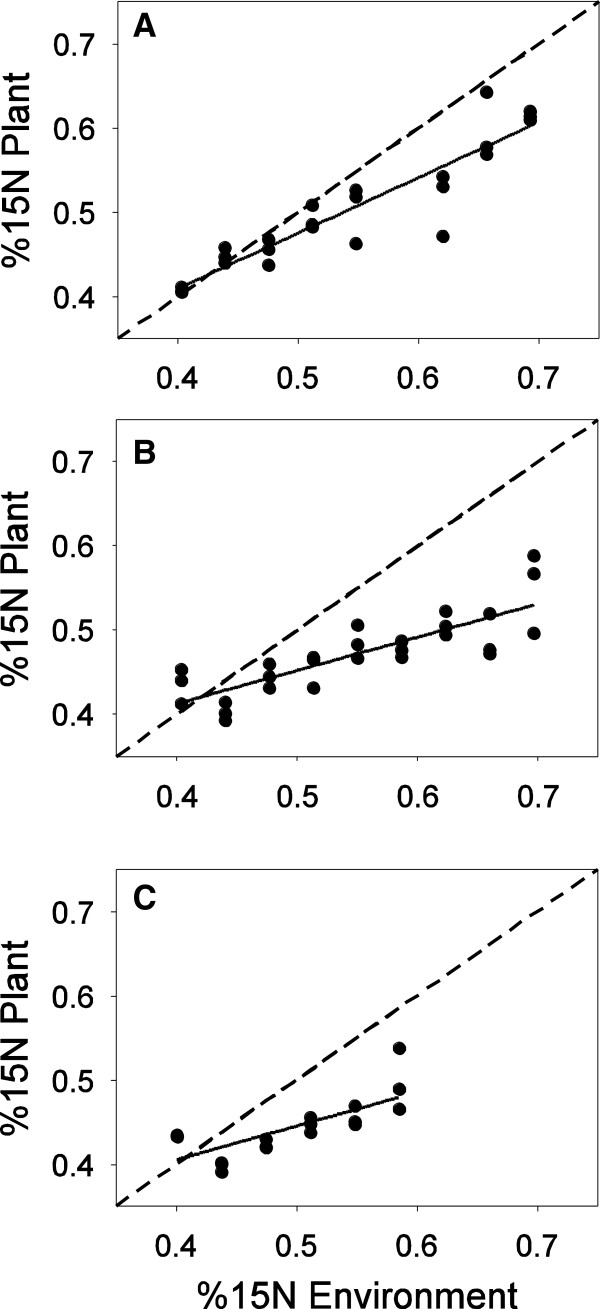
**Percent 15N available in the nutrient solutions versus percent 15N present in the plants.** Deviations of actual uptake (solid lines) below the null expectation of no preference (1:1 dotted line) indicate that the plant preferentially captured the unlabeled nitrogen source. **(A)** Nitrate and 15glutamine: nitrate preferred. **(B)** Nitrate and 15asparagine: nitrate preferred. **(C)** Glutamine and 15asparagine, glutamine preferred. Some plants were so small in the glutamine and asparagine treatment and it was not possible to obtain stable isotopes measurements resulting in missing data for panel **(C)**.

Second, to compare the relative amounts of each nitrogen type captured, the known %^15^ N supplied in the growth media was compared to the measured %^15^ N in each plant using linear regressions (Figure 4). The key test here was whether the slope of available versus captured differed from 1, and whether it was greater than, or less than, 1. In each case, the slope of the observed relationship was significantly less than 1, for nitrate and glutamine (t_23_ = 8.11, p < 0.001, Figure 4A), nitrate and asparagine (t_26_ = 3.18, p = 0.004, Figure 4B) and glutamine and asparagine (t_17_ = 4.36, p < 0.001, Figure 4C). This indicated that the plants preferentially captured the unlabeled nitrogen source in each treatment. Nitrate was preferred in the nitrate + glutamine treatment (Figure 4A), and in the nitrate + asparagine treatment (Figure 4B), and glutamine was preferred in the glutamine + asparagine treatment (Figure 4C). From this it was concluded that plant preferences followed a consistent hierarchy of nitrate > glutamine > asparagine across all pairs of nitrogen (Figure 4). This matched the predicted preferences from the single nitrogen data in experiment 2 (Figure 2).

**Figure 5:**
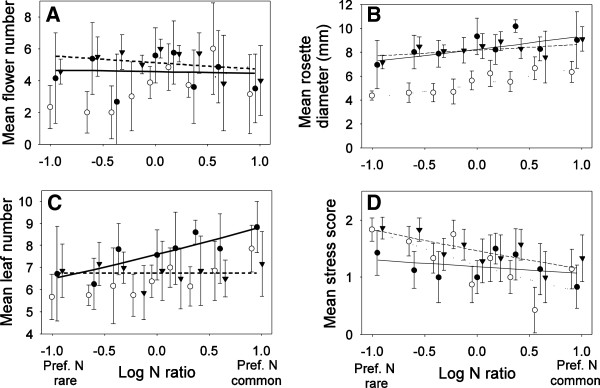
**Plot of log nitrogen ratio available in the environment, versus mean trait values for each pair of nitrogen from experiment 2 (1 Standard deviation is shown).** The fitted curves from the GLMs (Table 2) are also shown. In all panels, nitrate + glutamine (Filled triangles, solid line), glutamine + asparagine (open circles, dotted line) and nitrate + asparagine (filled circles, dashed line). Ratios corresponding to the preferred nitrogen type as either rare or common are indicated along the x-axis. **(A)** Flower number, **(B)** rosette diameter, **(C)** number of leaves, and **(D)** stress index. Note that count data was fit with a Poisson error distribution and a log link function, which can produce the appearance of non-linear fits in the untransformed scale.

Third, the influence of nitrogen mixtures and ratios on plant growth and performance was investigated. In this experiment there was always 1 mM of total nitrogen and only the ratio of nitrogen types offered was manipulated. As a result there was more overlap of plant traits among treatments which resulted in significant interactions among nitrogen types and ratio for most plant traits (Table 2). Significant interactions were recorded for flower number, rosette diameter and leaf number (Table 2, Figure 5A-C). These interactions were interpreted as stemming from the fact that plants performed similarly when nitrate was present regardless of which amino acid co-occurred with nitrate causing overlap of the regression lines between the nitrate + glutamine and nitrate + asparagine treatments (Figure 5, Table 2). It was also observed that, as the ratios available shifted in favour of the preferred nitrogen type, plant performance generally increased by all metrics, but the slopes were relatively shallow demonstrating that this increase was marginal (Figure 5).

**Table 2:** Results of GLMs on plant traits by nitrogen mixture, and nitrogen ratio in experiment 3

**Trait**	**Factor**	**Df**	**F**	**p**
Flower number	Nitrogen	2	12.1	<0.001
	Ratio	1	12.5	<0.001
	Nitrogen × Ratio	2	5.9	0.003
	Residuals	164		
Rosette diameter	Nitrogen	2	116.9	<0.001
	Ratio	1	33.2	<0.001
	Nitrogen × Ratio	2	4.3	0.015
	Residuals	164		
Leaf number	Nitrogen	2	14.2	<0.001
	Ratio	1	14.6	<0.001
	Nitrogen × Ratio	2	4.8	0.009
	Residuals	164		
Stress index	Nitrogen	2	2.8	0.06
	Ratio	1	12.2	<0.001
	Nitrogen × Ratio	2	1.8	0.166
	Residuals	164		

## Discussion

Increasingly, plant ecologists working at all levels of organization have become interested in the role of amino acid nutrition in the lives of plants [1-4,6,7,10,12,18]. Many authors report uptake of amino acids, but this uptake is only rarely linked to plant biomass production or potential reproductive effort of plants. Our data suggest that amino acids are relatively poor sources of nitrogen compared to nitrate, and that amino acids contribute a very small amount to growth and potential reproductive output in *A. thaliana* (Figure 2, Figure 5). Though *A. thaliana* plants in this study captured amino acids intact (Figure 3) and could grow and reproduce on both asparagine and glutamine as the sole nitrogen source (Figure 2), plants grown on amino acids were of often less than half the size of plants grown with nitrate as the only nitrogen source (Figure 2). Mixed nitrogen nutrition produced similar results, with the plants grown only on asparagine  + glutamine achieving only a fraction of the growth of plants supplied with nitrate and either amino acid (Figure 5). Plants also appear to preferentially capture nitrogen types following a consistent hierarchy of preferences, with nitrate > glutamine > asparagine (Figure 4).

Not only did plants grow poorly on amino acids, but the mixed nitrogen nutrition experiment revealed that the contribution of each nitrogen form was not additive. Instead, there appeared to be a negative interaction between different nitrogen types that was costly to the plant in terms of potential performance. For example, plants grown solely on nitrate in experiment 2 achieved the highest performance in any of our experiments, with a mean of 11.9 flowers and a mean rosette diameter of 15.1 mm at the highest concentration (0.9 mM NO_3_) of nitrate (Figure 2A,B). However, in experiment 3, with an identical concentration of nitrate plus additional nitrogen in the form of amino acids (0.9 mM NO_3_ + 0.1 mM amino acid N = 1 mM total nitrogen), plants achieved a mean of 3.5 flowers and a mean rosette diameter of only 9.0 mm. That is, even though the amount of nitrate was identical, and the total amount of nitrogen was actually higher, plants had only 29% as many flowers, and achieved only 75% of the vegetative growth with mixed nitrogen nutrition (Figure 5) compared to pure nitrate nutrition (Figure 2). This lower performance in the presence of similar nitrogen concentrations suggests that amino acid capture comes at some net-cost to growth and reproduction which outstripped any benefits that might be expected from increased access to nitrogen. These differences were not due to differences in calcium, as all three solutions contained an identical concentration of Ca(NO_3_)_2_. The differences among these treatments might have been due to differences in pH between solutions (0.9 mM NO_3_ pH 7.07; 9:1 NO_3_:Gln pH 6.67; 9:1 NO_3_:Asn pH 6.80), however with a difference of ±0.4, this pH range was within the margin of error of many experiments that attempt to control pH.

Plants grew significantly worse on asparagine compared to glutamine (Table 1, Figure 2). This might have been due to differences in uptake kinetics. These uptake kinetics are well described for the model plant *A. thaliana*. At concentrations similar to the range used in the present experiment, *A. thaliana* has almost identical uptake rates for both glutamine and asparagine (~1 umol g^-1^ DW h^-1^, [4]). Despite the nearly equal maximum influx rates, and the fact that both amino acids contain two nitrogen atoms, the results presented here show that these two amino acids are not equal in terms of their contribution to plant growth and reproduction (Figure 2, Figure 5). In all experiments vegetative traits related to potential reproductive output were significantly lower when plants were grown with asparagine compared to glutamine. This suggests that uptake of asparagine may result in some unknown fitness cost compared to glutamine in terms of uptake, transport and/or metabolism. Additionally, even though asparagine and glutamine have potentially equal maximum influx rates when offered singly [4], when we offered the two amino acids in mixture, the plants in this study preferentially captured glutamine over asparagine. Since influx rates and affinities are similar for glutamine and asparagine [4], we expected *A. thaliana* to capture these two amino acids in identical amounts. However, this was not what we observed; instead the plants somehow preferentially captured glutamine over asparagine (Figure 4c). This preferential uptake suggests that influx rates are not constant and plants may modulate their nitrogen uptake when provided with multiple types of nitrogen (Figure 4C). For example, Tomato (*Solanum lycopersicum*), is capable of modulating the expression of genes related to nitrogen uptake such that uptake sites are only expressed for those nitrogen types that are available [19]. These genes in *A. thaliana* and Tomato are homologous [19], supporting the idea that nutrient uptake in is neither passive, nor constant. However, most authors seem to assume that uptake is both passive and constant, even though data that might test this hypothesis are extremely limited. If plants do modulate their nitrogen uptake it may be part of a plant’s overall nitrogen foraging strategy in the same way that animals may choose prey that maximize benefits and minimize costs, plants may choose nitrogen types adaptively [20].

The standard ecological hypothesis for why plants capture amino acids, is that it enhances access to nitrogen, and presumably is a net-benefit to plants [1,2,7]. However, given that plants seem to grow poorly on amino acids and our results revealed that amino acids negatively interact with mineral sources to further reduce plant growth and reproductive potential this led us to ask: Why does *A. thaliana* bother to capture amino acids? There is clear evidence that plants do capture amino acids in field conditions [2,7,9,10,12,21], and many genes for uptake and transport of amino acids have been identified [1,4,18], indicating the potential for selection to act on the allelic variability in these genes associated with uptake. One hypothesis is that plant uptake is constant and hardwired, and plants simply have no “choice” in the matter. Plants simply capture whatever nitrogen sources are nearby in accordance with passive Michaelis-Menten kinetics. Though this seems to be prevailing wisdom, we reject this hypothesis, as evolutionarily unstable: any genes that do not offer a net benefit to plants will be removed from the population via natural selection, thus there must be some benefit to amino acid uptake that was hidden in our simple experiment. A second hypothesis is that there may be a pleiotropic effect where genes associated with nitrogen uptake are also linked with other phenotypic traits that are under stronger selection and thus sub-optimal uptake is maintained because of selection on other traits. There are several knock-out mutants for genes associated with nitrate uptake [18,22] and amino acid uptake [1,4] that might aid in the study of this question. If amino acid uptake genes are advantageous because of pleiotropy, we would expect a plant with genes associated with amino acid uptake to perform poorly compared to wild type plants even under pure nitrate nutrition. Finally, a third hypothesis is that, as in many aspects of plant nutrient foraging, the answer may be in competitive interactions among plants E.g. [7,9,23-28]. Specifically, that amino acid nutrition might not necessarily be an adaptation to enhance nitrogen uptake *per se*, but rather it might be an adaptation to enhance nitrogen uptake relative to potential competitors through resource partitioning. Evolutionary game theory has revealed that many aspects of plant-plant interactions produce evolutionary arms races that result in a tragedy of the commons [29], and this might be what happened in our experiment. That is, past competition for nitrogen might have produced character displacement causing plants to shift to less desirable nitrogen sources as a result of competition [30]. If this is true, the benefits of mixed nitrogen nutrition might only be apparent when plants face competition for nitrogen [23,24]. For example, plants preferentially place roots in nutrient rich soil patches, but the benefits of this behaviour only become apparent when plants compete for patches [23,24]. When plants grow alone, this root foraging behaviour often has no obvious benefits [31], and may even be costly [32]. This occurs because plants over-proliferate roots (sensu 25) in order to pre-empt competitor resource supply, but when competitors are absent this over-proliferation is not advantageous. Thus, one explanation for our results is that something similar occurs for amino acid uptake, where plants are attempting to partition resources with their competitors, but in the absence of competition this is not advantageous. However, there is currently limited data to examine this question.

Based on our results, and the lack of information about how uptake is altered by the presence of a competitor, two paths forward are suggested. First, more data are needed from ecologists about availability and importance of amino acids from a range of species and ecosystems. Much of the data surrounding uptake of amino acids comes from model plants and crop species. While we can certainly learn what is possible from model plants, studies of model plants do not provide information on the range and diversity of responses that may occur among plants in general. In terms of ecosystems, much of the data that suggests an important role of amino acids in shaping competitive and community outcomes among plants comes from relatively cool boreal, arctic and alpine systems where organic nitrogen is the dominant form in the soil [2,7-9,17,21,33-35]. However, the link between nitrogen preference and community composition has been much more elusive in more temperate systems, where decomposition of organic nitrogen occurs at a higher rate [10,36,37] and is rarely studied in tropical systems. Thus, while it is intriguing to think that amino acid uptake might form a major component of the ecology and evolution of plants E.g. [2,7], these data from arctic and alpine systems linking amino acids preferences to plant community composition might be the exception rather than the rule. The “acid test” for the generality of these ideas surrounding nitrogen partitioning for the ecology and evolution of plants worldwide will come from tropical and temperate systems where decomposition rates lead to higher turnover of organic pools, and higher microbial activity leads to more intense competition with microbes [1,10].

Second, more data are needed from plant ecophysiologists that examine the role of amino acid nutrition when plants compete and when they are offered mixed nitrogen sources. Key questions that have little data in the literature are: Do patterns of uptake change as a function of the presence or absence of competitors? And do patterns of uptake change in mixed versus single nitrogen solutions? Almost all experiments in the literature are based upon uptake of a single nitrogen type by plants that do not experience competition. However, there are a small number of studies that shed light on this process. For example, Miller et al. [10] used neighbour removals and ^15^ N tracers in the field to examine how neighbour identity influenced capture of ammonium, nitrate or glycine. They found a strong influence of neighbour identity in shaping the types and amounts of nitrogen captured [9]. This pattern might be caused purely by patterns of resource depletion by neighbours E.g. [38], but it might also be caused by plants adjusting their uptake kinetics in the presence of neighbours. In another study Abbes et al. [33] provided onion plants (*Allium cepa*) with nitrate and ammonium at a variety of ratios using a design that was similar to our experiment 3. They found that depletion trajectories of both nitrate and ammonium differed substantially based on the ratios of nitrogen available in the environment [39]. This supports the idea that uptake kinetics are labile and may be adjusted based on availability of nitrogen types in the environment. However, remarkably few data are available, and most studies do not provide multiple nitrogen types, adjust ratios, or examine the effects of competition. Given the few interesting results that are available [9,39], additional studies that track uptake kinetics and gene expression in mixed nitrogen nutrition experiments, and when plants compete would provide key information about the plasticity plants possess in uptake kinetics, and greatly advance the general understanding of plant nitrogen nutrition in all fields of plant sciences.

## Conclusions

It is now recognized that plants regularly capture amino acids along with mineral sources of nitrogen, and it is generally assumed that amino acid nutrition enhances access to nitrogen and is a net fitness benefit to plants [1,3]. The results presented above suggest that at concentrations that are similar to those found in natural soils, amino acids contribute relatively little to potential vegetative growth or reproductive output in *A. thaliana* relative to inorganic forms of nitrogen such as nitrate. In all experiments plants achieved lower performance when supplied with amino acids, and preferentially captured nitrate over amino acids when given nitrogen in mixture. Furthermore, there was a negative interaction between amino acids and nitrate when plants were fed with mixed nitrogen nutrition. These plants had lower overall growth and potential reproductive output then plants fed a similar amount of a single type of nitrogen. This suggests some cost associated with and expanded nitrogen diet, and raises questions about why plants bother to capture amino acids from soil. Faced with these curious results, we hypothesize that the importance of amino acid nutrition may be related to nitrogen competition, and this role may only be obvious in experiments that compare plants grown alone to those grown with neighbours. Regardless of the outcome, examining this untested hypothesis will provide valuable data about whether and how plants adjust their nutrient uptake kinetics.

## Methods

### Growth conditions

In all three experiments, individual *A. thaliana* plants were grown inside 50 mL culture tubes (Eppendorf) in a growth chamber (16:8 light:dark, 20°C, 180 μmol/m^2^/sec photosynthetic photon flux density, 18% relative humidity) for 4 weeks. Tubes were sealed with micropore surgical tape (3 M, St. Paul, Minnesota, USA) that prevented contamination but allowed gas exchange. A modified Hoagland’s solution recipe was used that contained no nitrogen so that all plants throughout the experiment received equal amounts of every nutrient except for nitrogen, the where the type and abundance of nitrogen were varied. The 1X N-free Hoagland’s solution contained: 5 mM K_2_SO_4_, 2 mM MgSO_4_, 0.5 mM KH_2_PO_4_, 4.5 mM CaSO_4_, 46.3 μM H_3_BO_3_, 0.76 μM ZnSO_4_, 0.32 μM CuSO_4_, 0.0025% (w/v) Iron Chelate (Plant Products Co. Ltd.), 0.66 μM NaMoO_4_[40]. Each plant was grown with 15 mL of the nutrient solution diluted to 0.1X, and 0.75% (w/v) of Phytablend, an agar based media (Caisson Laboratories, North Logan, Utah, USA). The mixture of N-free Hoagland’s solution and Phytablend was autoclaved for sterilization, and media was poured into sterile tubes within a laminar flow hood to maintain a sterile environment.

Nitrogen was added in three forms: (i) nitrate (Ca(NO_3_)_2_), (ii) asparagine (C_4_H_8_N_2_O_3_), and (iii) glutamine (C_5_H_10_N_2_O_3_). These were chosen because a previous study of amino acid uptake showed that *A. thaliana* grew best on the amino acids glutamine and asparagine compared to all other amino acids [4]. Thus, if glutamine and asparagine do not contribute significantly to plant growth and reproduction, other amino acids were expected to contribute even less. Nitrogen was added to the 0.1X N-free Hoagland’s solution at a concentration ranging from 0.1-1 mM, and we took into account the fact that each type of nitrogen had two nitrogen atoms per molecule when preparing solutions. For example, a concentration of *x* mM of each salt, contains a concentration of 2*x* mM total nitrogen. The nitrate salt will dissociate freeing two nitrate ions per molecule of nitrate salt. However, the amino acids will not dissociate. This means that plants will encounter amino acids half as often as they encounter nitrate ions, but each encounter with an amino acid yields twice as many nitrogen atoms as an encounter with a nitrate ion. Thus, plants have equal opportunity to capture all forms of nitrogen based on the number of atoms per molecule and the rate at which plants encounter each nitrogen type in the solution. Nitrogen solutions were not autoclaved but were filter sterilized through a 0.22 μm MF-Millipore MCE Membrane (Milex, Billerica, Massachusetts, USA), and added to the cooled sterile media within the laminar flow hood. The pH of all solutions was recorded (Additional file 1: Table S1).

It is common for physiologists to use pH buffered nutrient solutions because pH influences nitrogen uptake, and also to constantly refresh nutrient solutions to maintain steady-state nutrient concentrations. We consciously decided not to take this approach. While it is true that pH influences nitrogen uptake [5], it is also true that pH naturally covaries with nitrogen concentration, and that plants growing outside the lab likely do not experience pH buffered soil. Thus, since we were interested in ecological implications of nitrogen uptake, we allowed pH to naturally covary with nitrogen concentration, and viewed any shifts in pH associated with different types or concentrations of nitrogen as an intrinsic cost or benefit associated with capturing a specific type of nitrogen. Similarly, nutrients are often maintained at a steady-state concentration to avoid depletion in physiological studies that seek to measure Michaelis-Menten kinetics or other physiological processes because nutrient concentration effects influx rates. However, nutrients that are captured at a higher influx rate, must be refreshed more frequently and this would produce a situation where plants with different influx rates potentially have access to different amounts of total nutrients during the course of such an experiment. Because we were interested in comparing plant growth, and traits associated with plant performance we decided it was critical to control the total nutrients provided, rather than control the steady state concentration of nutrients. This is more ecologically relevant, where depletion by neighbours in soils drives many ideas about plant-plant competition [38,41]. We also point out that since nitrate was added as calcium nitrate, plants in the nitrate treatment had access to slightly more calcium (An additional 0.1-1 mM depending on the treatment, see below) compared to plants in the amino acid treatments. However, it is impossible to add nitrate without simultaneously adding some other positively charged ion, and it is impossible to add calcium without adding some other negatively charged ion. Since most ions influence nutrient uptake in some way [5] we accepted this as a limitation of the chemistry, while recognizing that it is not ideal. Our goal was to blend common physiological experimental tools (e.g. sterile media, *A. thaliana*), with more common ecological tools (natural covariance between nitrogen type and pH, a depletion experiment) to gain a clearer picture of the ecological importance of single and mixed nitrogen nutrition.

### Experiment 1: plant traits and reproductive effort

To link growth traits to potential lifetime reproductive effort, an experiment was performed where plants were grown in 1X Hoagland’s solution (as above) amended with 10 mM calcium nitrate as a source of nitrogen. To maximize the range of plant sizes for reproductive output-trait correlations the concentration of the whole Hoagland’s mixture was varied (i.e. 1X Hoagland’s as above + 10 mM Ca(NO_3_)_2_) by diluting the whole mixture to 0.001X, 0.034X, 0.067X, 0.101X or 0.135X. This concentration range was intentionally wide as we wanted the low end of the concentration range to stress plants enough that they had low reproductive output. Each concentration was replicated 25 times for a total of 125 plants. After 4 weeks of growth, the number of leaves and flowers were counted on each plant and plant stress was scored on a three point scale based on colour of leaves (0 – green leaves, not stressed, 1 – some yellow leaves, moderate nutrient stress, 2 – some red-purple leaves, high nutrient stress). Additionally, each plant was photographed individually through the transparent wall of the tube, at a constant distance and angle from the camera. From the images, rosette diameter (mm) was measured using ImageJ (v1.43, http://rsbweb.nih.gov/ij/) and measurements were calibrated for distance and angle by photographing a tube which contained a ruled paper disc, at the same location of the rosette inside a tube.

After trait measurement, plants continued to grow, and were permitted to fully senesce for 10 weeks. At senescence the seeds of each plant were collected into transparent trays. The seed crop of each plant was scanned individually and the particle counter in ImageJ was used to count seeds for each plant. Each trait at 4 weeks of age was correlated to seed production at senescence (10 weeks) linear regression in R to estimate a link between traits and reproductive output [42]. It was not possible to link biomass to reproductive effort because we needed to allow plants to continue to grow to senescence.

### Experiment 2: single nitrogen nutrition

In this experiment the effect of nitrogen type and concentration on plant growth and potential reproductive effort of *A. thaliana* plants was investigated. Plants in this experiment were grown for 4 weeks as described above, but were supplied with a single type of nitrogen (either calcium nitrate, glutamine or asparagine) at nine different concentrations between 0.1-0.9 mM (these values represent concentrations of nitrogen atoms, taking into account the fact that each nitrogen salt had two N atoms per molecule). Each treatment was replicated 8 times (3 nitrogen types × 9 concentrations × 8 replicates = 216 plants). The concentrations of every nutrient except nitrogen were held constant across all treatments. This allowed us to measure differences in growth and performance that were associated only with nitrogen type and concentration.

After 4 weeks of growth, plants were harvested, and rosette diameter, leaf number flower number and stress were measured. For each trait, individual GLMs were fit with trait as the dependent variable, and a factorial combination of nitrogen concentration (continuous) and nitrogen type (categorical) as fixed effects (R statistical Environment). Count data (Flower number, leaf number, stress score) was analyzed with a Poisson error distribution and a log link. Continuous data (rosette diameter) was log(x + 1) transformed for normality and analyzed with a Gaussian distribution of errors and an identity link (R statistical environment, [42]).

Some plants in the 0.6 mM nitrate treatment grew extremely poorly leading us to suspect there had been an error in preparation of the nutrient solutions, and thus this treatment was excluded from analysis.

### Experiment 3: mixed nitrogen nutrition

In this experiment, the effect of mixed nitrogen nutrition on plant growth and performance was examined. As above, plants were grown for 4 weeks, but were supplied with two types of nitrogen simultaneously at 9 different ratios. Ratios ranged from 0.1:0.9 mM to 0.9:0.1 mM in 0.1 mM increments (i.e. 9 ratios 1:9, 2:8, 3:7, 4:6, 5:5, 6:4, 7:3, 8:2, 9:1), but the total amount of combined nitrogen always summed to 1 mM (these values represent concentrations of nitrogen atoms, taking into account the fact that each nitrogen salt had two N atoms per molecule). Nitrogen types were combined in all pairwise combinations, (nitrate with glutamine, nitrate with asparagine, and glutamine with asparagine) and each treatment was replicated eight times (3 N-mixtures × 9 ratios × 8 replicates = 216 plants). Again, the amounts of the other nutrients were held constant across all treatments allowing us to measure only the effect of nitrogen types and ratios on plant growth.

To investigate relative amounts of each nitrogen type captured, in each treatment, one amino acid in each pair was dual labeled with ^15^ N and ^13^C. All carbon and nitrogen positions were labeled in each molecule. Nitrogen solutions were elevated to approximately double atmospheric abundance of ^15^ N (0.73% ^15^ N) while the second type of nitrogen remained at the atmospheric concentration of ^15^ N (0.37% ^15^ N). An aliquot of each labeled and unlabeled solution was saved and the exact isotopic ratios of each solution used in the experiment are given in Additional file 1: Table S2. In each case the target enrichment of 0.73% was achieved, with glutamine enriched to exactly 0.729% ^15^ N, and asparagine enriched to 0.733% ^15^ N in the nitrate/asparagine and glutamine/asparagine treatments. The target level of ^15^ N enrichment resulted in an enrichment of 1.508% ^13^C for glutamine and 1.493% ^13^C for asparagine (Additional file 1: Table S2).

After 4 weeks of growth, plants were harvested, and rosette diameter, leaf number, flower number and stress were measured. All aboveground biomass was thoroughly rinsed in deionized water and shoots were dried at 60°C. Three plants from every treatment were randomly selected for stable isotope analysis for a total of 81 plants (3 mixture treatments × 9 ratios × 3/8 sampled plants = 81 plants). The aboveground shoot of each plant was ground and both %^13^C and %^15^ N were determined for each plant using an Elemental Combustion System (Costech ECS 4010, Costech Analytical Technologies Inc.) coupled to a continuous flow isotope ratio mass spectrometer (Finnigan Delta Plus Advantage, Thermo Finnigan, Bremen Germany). Isotopic analysis is described in detail in Additional file 1. Roots were not included, because it was not possible to extract roots from the agar in a way that would not bias the isotopic analysis.

Dual labeled amino acids were used to ensure that amino acids were captured intact by correlating the %^15^ N and %^13^C content of each plant. A positive relationship between %^15^ N and %^13^C would indicate that amino acids were captured intact. To examine how much of each nitrogen type was captured the %^15^ N available in the nutrient media was correlated with %^15^ N present in the plants by linear regression. Because only one nitrogen type was labeled, any depletion or enrichment of plant ^15^ N relative to the environment was entirely due to preferential uptake of either the unlabeled or the labeled nitrogen type respectively. Three outcomes were possible: (i) if plants had no preferences the regression line would have a slope of 1, indicating that both types of nitrogen were simply captured in proportion to availability. Alternatively, (ii) lower plant %^15^ N compared to available would indicate preferential uptake of the unlabeled nitrogen, and (iii) higher plant %^15^ N it would indicate preferential uptake of the labeled nitrogen.

As above, plant traits were analyzed using general linear models. In this analysis plant trait was included as a dependent variable, with a factorial combination of nitrogen mixture (categorical, nitrate + glutamine, nitrate + asparagine, glutamine + asparagine) and the log ratio of nitrogen availability (continuous, e.g. 0.1 mM:0.9 mM = log(1/9) = −0.95) as fixed effects.

Plants in the glutamine/asparagine treatment grew very poorly and were often too small to obtain enough tissue for stable isotope analysis, and thus isotope data was available only for some plants from this treatment. Additionally, all tubes in the nitrate/glutamine treatment at a ratio of 0.4 mM:0.6 mM became severely contaminated with bacteria, and thus this treatment was excluded.

## Competing interests

The authors declare that they have no competing interests.

## Authors’ contributions

GGM, JFC and MKD conceived of the study, and participated in its design and coordination and helped to draft the manuscript. GGM performed the experiments. GGM and JFC performed the statistical analyses. All authors read and approved the final manuscript.

## Supplementary Material

Additional file 1 **Calculation of stable isotope ratios. Table S1:** Initial pH of each nutrient solution used in experiment 2 (single nitrogen nutrition: top) and experiment 3 (mixed nitrogen nutrition: bottom) for each type or ratio of nitrogen, and each abundance or ratio of nitrogen. **Table S2:** Atomic percentages of ^15^N and ^13^C in the enriched and un-enriched nitrogen solutions used in the experiment. Aliquots of each solution used to grow the plants were saved and analyzed in triplicate in the same way as plant tissue. Click here for file
