# Supplementary material for: Ecological implications of single and mixed nitrogen nutrition in Arabidopsis thaliana
Source: BMC Ecol. 2013 Jul 23;13:28. doi: 10.1186/1472-6785-13-28 (PMC3723926; doi:10.1186/1472-6785-13-28)
Supplement: Additional file 1 — Calculation of stable isotope ratios. Table S1: Initial pH of each nutrient solution used in experiment 2 (single nitrogen nutrition: top) and experiment 3 (mixed nitrogen nutrition: bottom) for each type or ratio of nitrogen, and each abundance or ratio of nitrogen. Table S2: Atomic percentages of 15N and 13C in the enriched and un-enriched nitrogen solutions used in the experiment. Aliquots of each solution used to grow the plants were saved and analyzed in triplicate in the same way as plant tissue. [file 1472-6785-13-28-S1.docx]

# How important is amino acid uptake for plants? Linking uptake with fitness in *Arabidopsis thaliana*.

Gordon G. McNickle, Michael K. Deyholos, and James F Cahill Jr.

### **Additional Information**

*Calculation of stable isotope ratios.*

The isotopic enrichment of the samples (*dI*) was calculated for both ^13^C/^12^C and ^15^N/^14^N from the following equation:

$$dI=1000\left(\frac{R_{sam}}{R_{sta}}-1\right)$$

where *R_sam_* and *R_sta_* are the percentage of either d^13^C/d^12^C or d^15^N/d^14^N in the sample and standard respectively. For carbon, National Institute of Standards and Technology (NIST, Gaithersburg, MD, USA) standards of NBS 19, NBS 22 and LSVEC were used to calibrate internal standards of BMO, Corn Stover, Pea Grain Protein, and Red Clover which were used as the working standards. The internal standards had carbon isotope composition of –23.91‰, –12.5‰, -24.68‰, –27.32‰, and –27.42‰ relative to international standards (Pee Dee Belemnite), respectively. For nitrogen, NIST standards of IAEA-N1, IAEA-N2 and IAEA-N3 were used to calibrate the same internal standards as above. Standards had a nitrogen isotope composition of 8.2‰, 9.1‰, 2.429‰, 6.164‰, and -0.56‰ relative to international standards, respectively. Atomic percentage (%^13^C or %^15^N) was calculated from isotopic enrichment (d^13^C or d^15^N) based on known composition of the standards, and the estimated *dI* values.

| **Additional file 1: Table S1:** Initial pH of each nutrient solution used in experiment 2 (single nitrogen nutrition: top) and experiment 3 (mixed nitrogen nutrition: bottom) for each type or ratio of nitrogen, and each abundance or ratio of nitrogen. | | | |
| --- | --- | --- | --- |
| **Concentration**  **(mM)** | **Nitrate** | **Glutamine** | **Asparagine** |
| 0.1 | 7.02 | 7.31 | 7.35 |
| 0.2 | 7.00 | 7.63 | 7.33 |
| 0.3 | 7.02 | 7.67 | 7.30 |
| 0.4 | 6.98 | 7.61 | 7.30 |
| 0.5 | 7.00 | 7.68 | 7.28 |
| 0.6 | 6.91 | 7.67 | 7.24 |
| 0.7 | 7.04 | 7.63 | 7.22 |
| 0.8 | 6.96 | 7.59 | 7.20 |
| 0.9 | 7.07 | 7.59 | 7.19 |
| **Ratio** | **Nitrate & glutamine** | **Nitrate & asparagine** | **Glutamine & asparagine** |
| 1:9 | 6.98 | 7.13 | 7.22 |
| 2:8 | 6.95 | 7.02 | 7.19 |
| 3:7 | 6.89 | 6.91 | 7.19 |
| 4:6 | 6.85 | 6.85 | 7.15 |
| 5:5 | 6.83 | 6.82 | 7.13 |
| 6:4 | 6.80 | 6.80 | 7.11 |
| 7:3 | 6.82 | 6.78 | 7.11 |
| 8:2 | 6.82 | 6.80 | 7.11 |
| 9:1 | 6.67 | 6.80 | 7.13 |

| **Additional file 1: Table S2:** Atomic percentages of ^15^N and ^13^C in the enriched and un-enriched nitrogen solutions used in the experiment. Aliquots of each solution used to grow the plants were saved and analyzed in triplicate in the same way as plant tissue. | | | |
| --- | --- | --- | --- |
| Enrichment | Nitrogen | N15 | C13 |
| Unlabeled | Nitrate | 0.367 | - |
|  | Glutamine | 0.364 | 1.128 |
|  | Asparagine | 0.368 | 1.114 |
| Labeled | Glutamine | 0.729 | 1.508 |
|  | Asparagine | 0.733 | 1.493 |
